# Delayed presentation of lower cervical facet dislocations: What to learn from past reports?

**DOI:** 10.1051/sicotj/2023036

**Published:** 2024-01-18

**Authors:** Laurent Nkurikiyumukiza, Alex Mathias Buteera, Mohammad Mostafa El-Sharkawi

**Affiliations:** 1 University of Rwanda College of Medicine and Health Sciences P.O. Box 3286 Kigali Rwanda; 2 Faculty of Medicine, Assiut University Assiut 71515 Egypt

**Keywords:** Delayed presentation, Neglected, Lower cervical facet dislocations, Anterior approach, Posterior approach

## Abstract

Delayed presentation of lower cervical facet dislocations is uncommon, and there is no standardized way to approach these neglected injuries. The literature on neglected lower cervical facet dislocations is limited to case reports and few retrospective studies. This justifies the need for a comprehensive review of this condition. Our purpose was to elaborate a review on the epidemiology, clinical and radiological presentation, and treatment techniques and approach to these neglected injuries. Middle-aged adults from 30 to 50 represent 73.8% of reported cases, and most of them are males (72.0%). The most affected level is C5–C6 (43.0%). While most delays are due to missed injuries (52.1%) and ineffective non-operative treatment (36.2%), the other reason for delay is negligence in seeking medical care (11.7%). Patients present with variable degrees of neurological deficit, persistent neck pain, and neck stiffness. Reported approaches and techniques to reduce and stabilize these injuries are highly variable and depend on the surgeon’s judgment, experience, and preference. Fibrotic tissues and bony fusion around the dislocated facet joint contribute to the reduction challenge, and 77.0% of closed reduction attempts fail. Anterior and posterior approaches to the cervical spine are used selectively or in combination for surgical release, reduction, and stabilization. Despite the lack of standardized treatment guidelines and different approaches, most of the authors reported improvement in pain, balance, and neurology post-surgery. Starting with the posterior surgical approach aims to achieve reduction compared to the anterior approach which largely aims at spinal decompression. Given the existing controversies, the need for quality prospective studies to determine the best treatment approach for lower cervical facet dislocations presenting with delay is evident.

## Introduction

Lower cervical facet dislocations are unstable injuries that affect the anterior and posterior columns of the cervical spine [1]. Allen-Ferguson classified lower cervical facet dislocations as flexion-distraction injuries, which are further subdivided into flexion sprain, unilateral facet dislocation, bilateral facet dislocation with up to 50% displacement (perched facets), and complete dislocation with >50% displacement [1, 2]. They are associated with variable degrees of spinal cord injury (SCI) [3, 4]. Lower cervical facet dislocations that present before 3 weeks are considered acute while those presenting after 3 weeks are considered old, chronic, or neglected [5–7].

**Figure 1:**
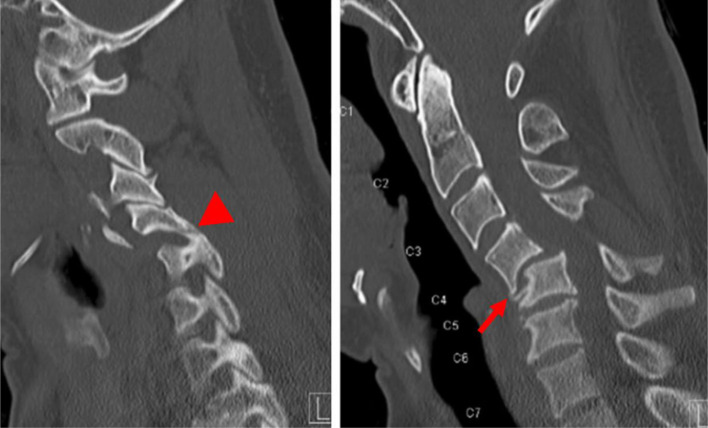
C-spine CT-scan sagittal slices image demonstrating a neglected C6–C7 facet dislocation. Note the osteocartilaginous tissues bridging C6 and C7 (red arrow) and locking of facets (red arrowhead).

While closed reduction for acute injuries is often successful, for lower cervical facet dislocations presenting with a delay beyond three weeks, fibrotic tissues and bony fusion around the injured facets and uncovertebral joints represent a challenge to reduction [7, 8] (Figure 1). The reported success rate of closed reduction for acute injuries cannot be replicated to neglected cases [9, 10]. Neglected injuries require an adequate surgical release for reduction [11, 12]. There is no established standard treatment approach for neglected lower cervical facet dislocations owing to the rarity of cases and lack of quality studies to guide treatment decision-making. The literature on neglected lower cervical facet dislocations is limited to case reports and few retrospective studies. A comprehensive review of this condition is lacking. We reviewed 20 articles accounting for 165 patients with neglected lower cervical facet dislocations published in the English literature, and we elaborated a comprehensive synthesis of the epidemiology, clinical and radiological presentation, and variable treatment techniques and approaches to these neglected injuries. The overview of cases reported in the literature is portrayed in Table 1.

**Table 1:** Characteristics of published cases with neglected lower cervical facet dislocations.

Variable	*n*	%
Age (16 articles/65 cases)		
Children (<12 years)	0	0.0%
Adolescent (13–17 years)	0	0.0%
Young adults (18–29 years)	4	6.2%
Middle adults (30–60 years)	48	73.8%
Older adults and elderly (>61 years)	13	20.0%
Sex (19 articles/150 cases)		
Male	108	72.0%
Female	42	28.0%
Affected level (20 articles/165 cases)		
C2–C3	1	0.6%
C3–C4	8	4.8%
C4–C5	39	23.6%
C5–C6	71	43.0%
C6–C7	41	24.8%
C7–T1	5	3.0%
Cause of delay (18 articles/94 patients)		
Missed injury	49	52.1%
Ineffective non-operative treatment	34	36.2%
Delay to seek medical care	11	11.7%
Type of dislocation (19 articles/150 cases)		
Unilateral	51	34.0%
Bilateral	99	66.0%
Mechanism of injury (16 articles/130 cases)		
Fall	64	48.5%
Road traffic accident	38	29.2%
Sport injury	9	6.9%
Heavy object fall on the head	20	15.4%
Clinical presentation (20 articles/165 cases)		
Neck pain	61	37.0%
Stiffness	50	30.3%
Deformity	2	1.2%
Neurological deficit	139	84.2%
Asymptomatic	2	1.2%
Neurological status on admission (19 articles/165 cases)		
Frankel A	20	12.1%
Frankel B	17	10.3%
Frankel C	41	24.8%
Frankel D	40	24.2%
Frankel E	26	15.8%
Radiculopathy	21	12.7%
SCI outcomes ≥6-month follow-up (16 articles/77 cases)		
Remained neurologically free	17	22.1%
Improved ≥2 Frankel grades	19	24.7%
Improved 1 Frankel grade	32	41.6%
No improvement–no deterioration	8	10.4%
Deterioration	1	1.3%

## Epidemiology

Around 74% of patients with lower cervical facet dislocations with a delay beyond three weeks occurred in middle-aged adults (30s–50s) and most series, male patients are 2–3 folds compared to females [5, 7, 13–16]. The most affected levels are C5–C6 (43.0%) and C6–C7 (24.8%), while C2–C3 and C3–C4 are the least affected [7, 13–16]. Most of these injuries are initially missed or managed inadequately with non-operative measures [5, 7, 14, 15, 17]. In reviewed articles, missed injuries and those treated with ineffective non-operative options accounted for 52.1% and 36.2% respectively. Cervical spine injuries are missed due to incomplete clinical and radiological assessment, especially for multiply injured patients [7, 18–21].

Other factors leading to the delayed presentation of lower cervical injuries include poverty, negligence, and the lack of resources (skilled surgeons, diagnostic and therapeutic infrastructures) [13]. Among reported cases, 11.7% of patients with mild to moderate symptoms did not seek medical attention and consulted later when symptoms persisted or complications arose (Table 1).

## Clinical presentation

Neurological deficits, persistent neck pain, and neck stiffness are common complaints from patients with neglected lower cervical facet dislocations [5, 7, 12–14, 16, 21]. Most patients report previous trauma events (falls, road traffic accidents, sports injuries, or heavy objects falling on their heads) [7, 14, 16, 22]. Few patients present with gross deformity [11, 23]. Neurological deficits range from minor to complete quadriplegia [7, 13, 16]. Some patients with neglected lower cervical facet dislocation have no neurological deficit [11, 12, 24, 25] or present isolated radicular involvement [5, 13, 14]. Reviewed articles on patients with neglected lower cervical facet dislocations revealed that 84.2% of cases had neurological deficits and at least 30% presented with persistent neck pain and/or neck stiffness (Table 1).

Detailed history, including previous treatments, is the key to understanding the patient’s complaints, general health status, and expectations. The examination of the neck checks for deformity and patient-controlled range of motion. The neurological examination evaluates sensory loss, muscle weakness, spasticity, hyperreflexia, abnormal plantar reflexes, and bowel/bladder function [13]. Frankel grading [26] and ASIA impairment scale [27] are widely accepted to describe the neurological status.

## Imaging

**Figure 2:**
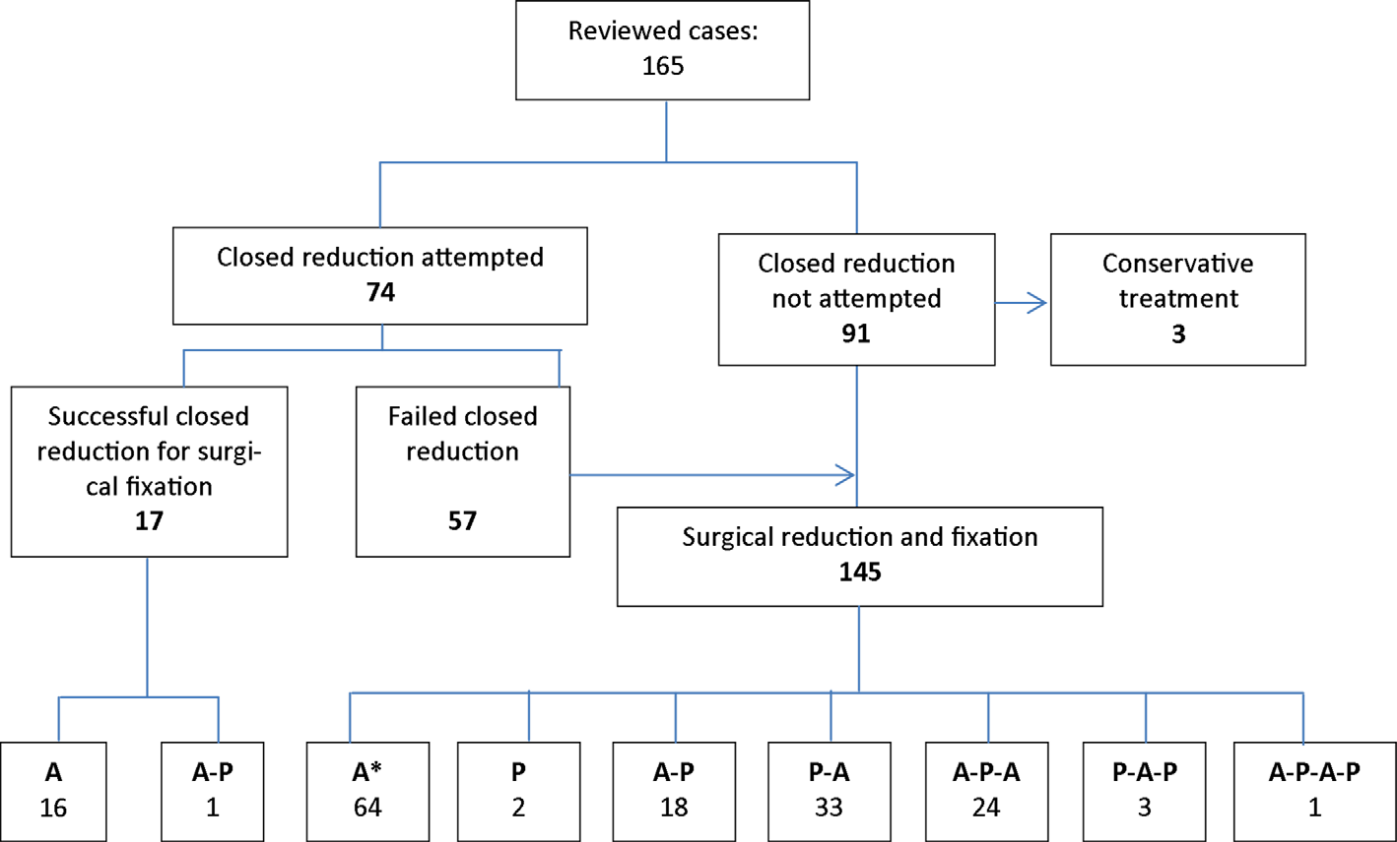
C-spine CT imaging showing neglected dislocation C4–C5. Note the anterolisthesis C4–C5 (red arrow) and bony fusion of the facet (red arrowhead).

For neglected lower cervical facet dislocations, imaging is essential for diagnosis and surgical planning [12, 15]. The goal of imaging is to delineate the affected level, the alignment and stability, the extent of cord compression, eventual herniated disc fragments, and other changes related to trauma or degenerative process [8, 11, 12, 15, 28]. Plain radiographs and computed tomography (CT) demonstrate variable degrees of anterolisthesis, sagittal malalignment, incongruent facet joint, and increased interspinous distance [29]. These modalities help to rule out associated fractures of facets, endplates, lamina, or spinous processes that influence the planning in of terms approach, reduction, and fixation technique [30, 31]. Patient-controlled dynamic views help to differentiate between stiff and flexible deformities [6]. The CT scan provides a three-dimensional pattern of the injury and delineates eventual bony fusion that requires surgical release before reduction [7, 12] (Figure 2).

The Magnetic Resonance Imaging (MRI) scan is the imaging of choice to assess the eventual disc herniation, spinal canal narrowing, spinal cord compressionand signal changes, and posterior ligamentous complex injury [13, 15]. CT myelogram is an alternative to MRI when the latter is contraindicated [3].

## Treatment goals and expectations

The treatment for neglected lower cervical spine consists of delicate major surgical procedures, often in multiple stages [8, 13, 30, 32]. The core treatment objectives are the decompression of neural elements, restoring stability and alignment, and achieving solid fusion [8]. Neck pain and malalignment significantly improve after successful surgical stabilization and fusion [5, 7, 14, 22, 33]. Full recovery of SCI is possible but cannot be guaranteed as most of these injuries yield variable neurological outcomes post-surgery, with the majority improving by one or two Frankel grades [5, 7, 13, 16]. In addition, neurological deterioration is possible [17]. Tables 1 and 2 portray the neurological outcome for reported cases. Complications and events to anticipate include bleeding, prolonged hospital stay, need for ICU admission, wound complications, pseudoarthrosis, and fixation failure (Table 3). When surgical risks outweigh the benefits, conservative treatment is preferred [11, 34].

**Table 2:** Neurological outcomes for patients with SCI after at least 6 months follow-up.

	Postoperative neurological status (Frankel grade) – 16 articles/77 cases				
Preoperative neurological status (Frankel grades)	A	B	C	D	E
A	4	0	1	0	0
B	0	1	4	6	1
C	0	0	1	10	11
D	0	0	0	2	18
E	0	0	0	1	17

**Table 3:** List of publications and approaches to neglected lower cervical facet dislocations.

Authors	Year	Cases	Mean age (years)	Mean delay (weeks)	Closed reduction (success/tried)	Approach	Fixation technique	Comments
Bartels and Donk [32]	2002	3	71.3	>10	0/0	P–A–P(2), A–P–A–P(1)	LMS, IBC, HP	Authors recommend starting with the posterior approach.
Bechet et al. [36]	2022	1	70	52	1/1	A	IBC, HP	Neurology improved after surgery from A to E.
Ding et al. [7]	2007	17	45.2	13.7	0/3	A–P	LMS, IBG	Use of interbody cancellous graft and LMS. Hospital stay: 7–30 days. Wound complications (2 cases), dura tear (1 case).
Hassan [5]	2002	12	50	>14	2/12	A(2), P(1), P–A(9)	HP or LMS	Traction after posterior release, hospital stays 15–29 days. One case of delayed union.
Jain et al. [22]	2010	4	48.2	11.75	0/1	P–A	PW, IBG, HP	Posterior spinous wiring process wiring. Good outcome.
Jiang et al. [15]	2013	14	46.9	20	3/9	A(3), A–P(1), A–P–A(10)	PW and HP or LMS and HP	Use of neuro-monitoring, no complication, all cases fused, surgery duration: 85–215 m. Hospital stay: 13.1 ± 4.6 days.
Liu et al. [14]	2011	9	45	>11	0/2	P–A	PW, IBC, HP	Cage subsidence (1 case), loss of reduction (1 case), and overall neurological improved and achieved fusion.
Marasini [30]	2020	3	48	>12	0/3	A–P–A	IBC, HP	Corpectomy of the distal vertebra (2 cases), in situ fixation (one case). Neurology improved in all cases. One case developed acute renal failure post-surgery.
Payer and Tessitore [8]	2007	1	51	10	0/0	A–P–A	IBC, HP, LMS	Blood loss: 530 ml fusion was achieved in all cases. Surgery duration: 4 h.
Prabhat et al. [13]	2017	15	33	9	5/15	A(4), A–P(1), A–P–A(9)	IBG, HP, LMS	Blood loss: 470 ml. ICU admission (4 cases), Need of tracheostomy (1 case). All patients improved neurologically.
Rajasekaran et al. [23]	2011	1	49	>8	0/1	P	LMS	Use of navigation and neuro-monitoring. Good outcome.
Shimada et al. [37]	2013	1	76	8	0/0	P–A	LMS, HP	Neurological improvement, fusion achieved.
Srivastava et al. [12]	2014	1	42	>60	0/0	P–A–P	LMS, IBC, HP	Surgery duration >5 h, no complication.
Srivastava et al. [6]	2016	6	51	6	3/6	A(3), P(2), P–A(1)	IBG, HP, PW or LMS	Prolonged ICU admission need of tracheostomy (1 case).
Thompson and Hohl [11]	1978	1	72	46	0/0	–	–	Fused, moderate deformity. Conservative treatment.
Jain et al. [25]	2020	1	26	>14	0/0	P–A	LMS, IBC,HP	Good surgical outcome.
Lukhele [33]	2005	15	24–56	>6	3/15	A	IBC, HP	ACDF for reduced cases. Corpectomy and fusion in situ for others. No deterioration, alignment improved, all fused.
Goni et al. [17]	2013	6	36	>8	0/6	P–A	HP alone or with PW	One patient had neurological deterioration; two patients became unstable during the operation.
Miao et al. [16]	2017	52	42.8	8.6	0/0	A	IBC and HP	Corpectomy and fusion in situ. Alignment and neurology improved. Fusion was achieved in all cases.
Shah et al. [34]	2018	2	30	>150	0/0	–	–	Moderate deformity and they were treated conservatively.

IBC: interbody cage, HP: H-plate, PW: posterior wiring, LMS: lateral mass screws, IBG: interbody graft, A: Anterior approach, P: Posterior approach, A–P: Anterior–Posterior, A–P–A: Anterior–Posterior–Anterior; P–A: Posterior–Anterior, P–A–P: Posterior–Anterior–Posterior, A–P–A–P: Anterior–Posterior–Anterior–Posterior.

## Treatment techniques and approaches

### Closed reduction and surgical stabilization

**Figure 3:**
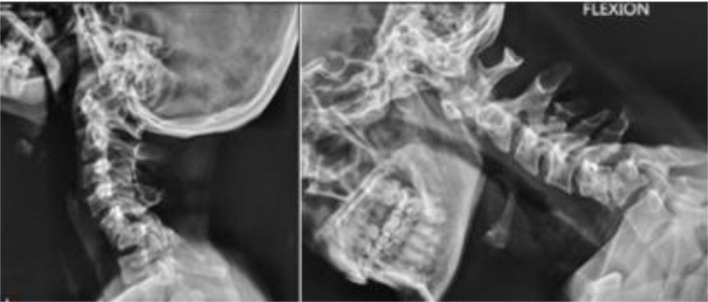
Summary of approaches used neglected lower cervical facet dislocations. (*cases underwent corpectomy and in situ fixation; A: Anterior; P: Posterior; A–P: Anterior–Posterior; A–P–A: Anterior–Posterior–Anterior; P–A: Posterior–Anterior; P–A–P: Posterior–Anterior–Posterior; A–P–A–P: Posterior–Anterior–Posterior).

Closed reduction for lower cervical facet dislocations requires craniocervical traction using Gardner-Well or Crutchfield tongs under conscious sedation [35]. It is indicated for alert and cooperative patients [10]. The success rate of closed reduction for acute lower cervical facet dislocation is approximately 80% [10] and decreases to 20% for delays beyond 72 h [9]. For lower cervical facet dislocations presenting after 3 weeks, closed reduction is likely to fail [5, 7, 13, 15, 17]. We recorded 77.0% failure of closed reduction attempts (Figure 3).

Despite the high failure rate, there was no neurological deterioration related to this procedure. Some authors avoided closed reduction attempts in cases with severe spinal canal narrowing or disc herniation [8, 15]. After successful closed reduction, most cases undergo anterior cervical discectomy and fusion [5, 6, 13, 15, 36].

### Posterior (P) and Posterior–Anterior (P–A) surgical approaches and techniques

The posterior approach to the cervical spine aims at releasing fibrotic and osteocartilaginous tissues around dislocated facet joints [5, 6, 12, 17, 22, 25, 32, 37]. After effective release, the techniques for reduction include levering the facet [15, 22], distracting the spinous processes [7], or craniocervical traction assistance [14]. Partial facetectomy is helpful when facets cannot be unlocked [5, 6, 14, 22]. After successful reduction, posterior fixation with lateral mass screws follows [6, 23]. Spinous process wiring is another alternative especially when the reduction does not require facetectomy [6, 14, 22, 38]. Benzel–Kesterson [39] and Rogers [40] techniques are widely used for spinous process wiring. Fractures of the spinous process or lamina are contraindications to this technique [31]. Adequate bone decortication and grafting before wound closure optimize conditions for fusion [23].

The use of isolated posterior fixation, though uncommon, has been reported [5, 6, 23]. In case of failure to achieve reduction, or when more stability is desired, the additional anterior procedure is warranted [6, 14, 17, 22, 25, 37]. After posterior wound closure, the patient is gently flipped in the supine position for the anterior surgery consisting of cervical discectomy to decompress neural tissues [12, 14]. Caspar distractor and interbody distractor are used to complete the reduction [7, 41]. Fluoroscopy images confirm the reduction before the final fixation with an interbody cage or iliac bone graft, supplemented by anterior plating [6, 14, 17, 22, 25, 37].

### Anterior (A) and Anterior–Posterior (A–P) surgical techniques

Lower cervical facet dislocations are associated with a high rate of intervertebral disc disruption and herniation [10]. Though the posterior approach is preferred for irreducible facet dislocations, the anterior approach is ideal for decompression of neural tissues, especially in cases with disc herniation [8, 12]. After discectomy, caspar distractor, interbody distractor, or craniocervical traction help to obtain reduction which is confirmed with fluoroscopy before the final fixation with an interbody cage or iliac bone graft, supplemented by anterior plating [6–8, 41]. Anterior cervical corpectomy and strut cage fixation is another option for cases with fractured vertebral endplates and those with inaccessible disc fragments [28, 30].

**Figure 4:**
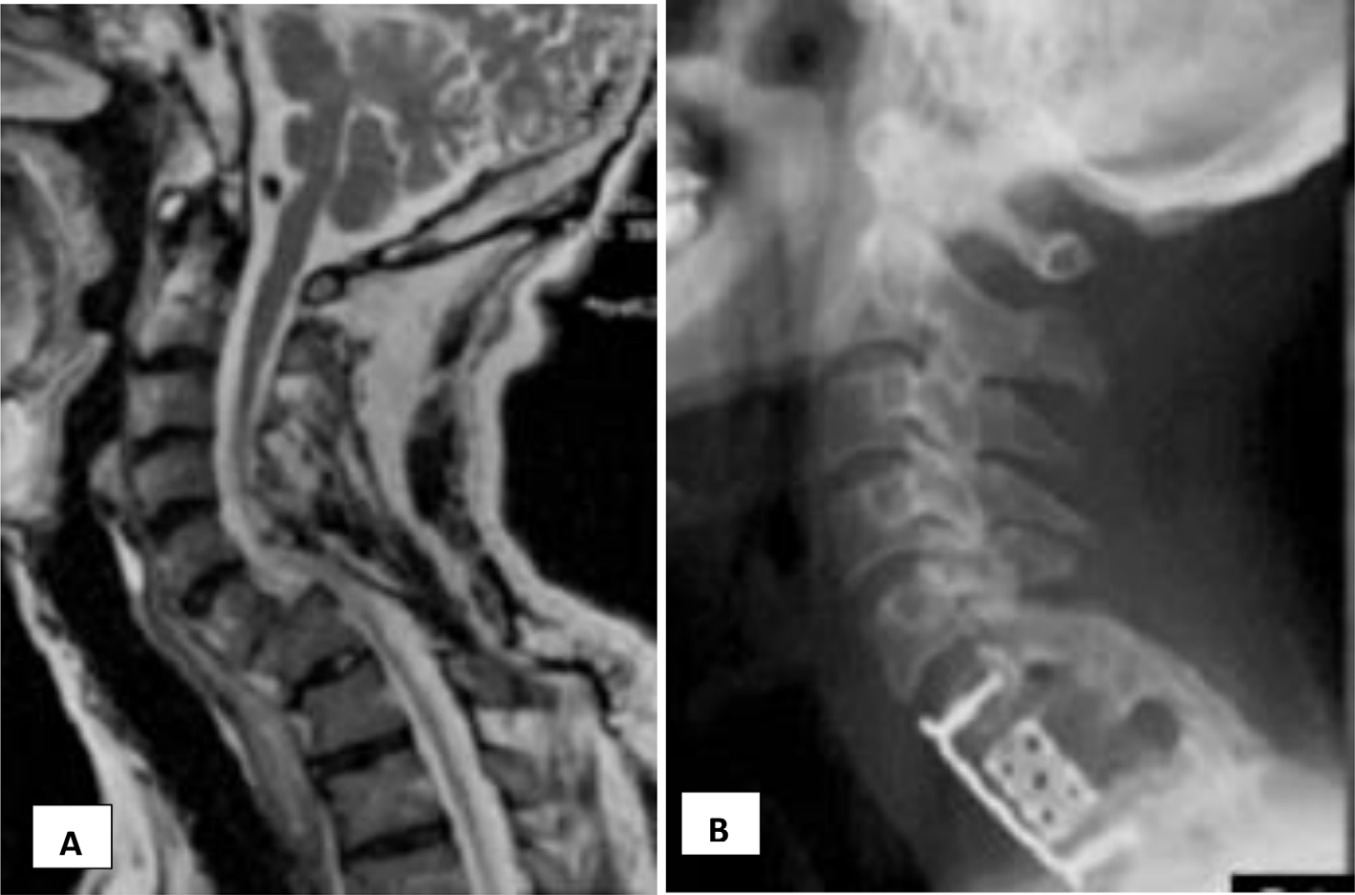
Neglected dislocation of C5–C6 (slide A). Reduction and combined anterior–posterior fixation (slide B).

When anterior reduction maneuvers fail, or more stability is desired, the posterior approach is warranted for further release reduction and fixation with lateral mass screws or spinous process wiring [7] (Figure 4).

### The need for more than two surgical procedures

Due to the reduction challenges, neglected lower cervical facet dislocations often required three or four procedures. Anterior–Posterior–Anterior (A–P–A) [8, 13, 15, 30], Posterior–Anterior–Posterior (P–A–P) [12, 32], and Anterior–Posterior–Anterior–Posterior (A–P–A–P) [32] procedures are reported in the literature. For all these cases, the first or second procedures did not achieve reduction and it required further procedures.

Failure to reduce the dislocation with the anterior procedure warrants a posterior approach for further release and reduction [8, 13, 15, 30]. In this scenario, a temporary interbody spacer cage helps to maintain stability while proceeding with the posterior surgical release, reduction, and fixation [8]. It requires a third anterior procedure to complete the anterior fixation and fusion [8, 13, 15, 30].

To avoid the third anterior procedure, surgeons tried different techniques [42–44]. Ding et al. [7] secured the cancellous bone graft in the interbody space with a gelatin foam and longus colli muscles, and proceeded with posterior surgery to reduce and fix dislocated facets with lateral mass screws.

In the same line, for cases where it was not possible to get the reduction through the posterior approach, they proceeded with the anterior decompression and further release to achieve the reduction, and it required a third posterior procedure to complete the posterior fixation [12, 32].

In general, anterior surgery for neglected lower cervical facet dislocations is aimed mainly at decompressing neural structures rather than reduction. For cases without disc herniation, authors advocated for starting with the posterior approach which, in most cases, achieved complete or near-complete reduction [5, 13, 14, 32]. The anterior approach is largely aimed at spinal decompression rather than reduction [8, 12].

These additional surgical procedures represent a burden and risk to patients, including but not limited to neurological deterioration, bleeding, radiation exposure, infection, airway complications, anesthesia complications, and cost. Longer operative time of more than 4 h and excessive blood loss were other drawbacks [8, 12, 13].

### Conservative treatment, partial reduction, and in situ fusion

**Figure 5:**
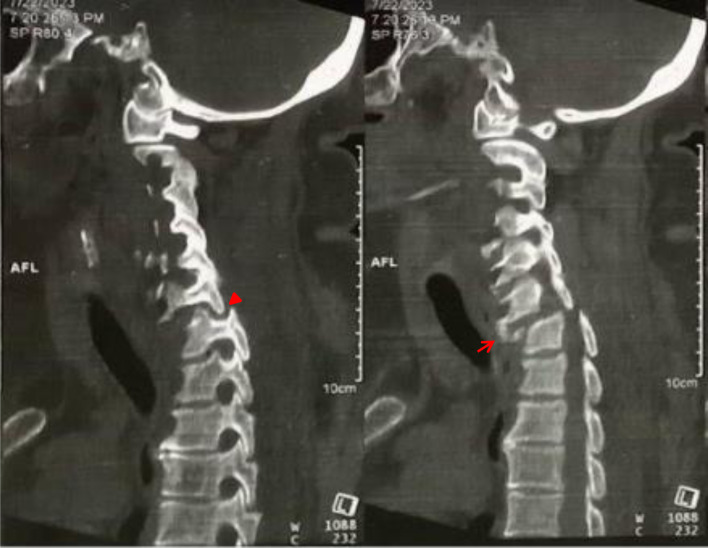
Dynamic radiographs of the cervical spine showing neglected dislocation C5–C6 treated conservatively. Note the stability of the dislocated segment and anterior auto-fusion.

Lower cervical facet dislocations presenting with delay are sometimes found incidentally after an unrelated event. Thompson and Hohl [11] treated conservatively an asymptomatic alcoholic patient with bilateral cervical facet dislocation who presented years after the index trauma. Shah et al. [34] reported 2 cases of neglected cervical facet dislocations that were managed conservatively. For all these cases, there was radiological fusion with moderate deformity, and without neurological deficit (Figure 5).

**Figure 6:**
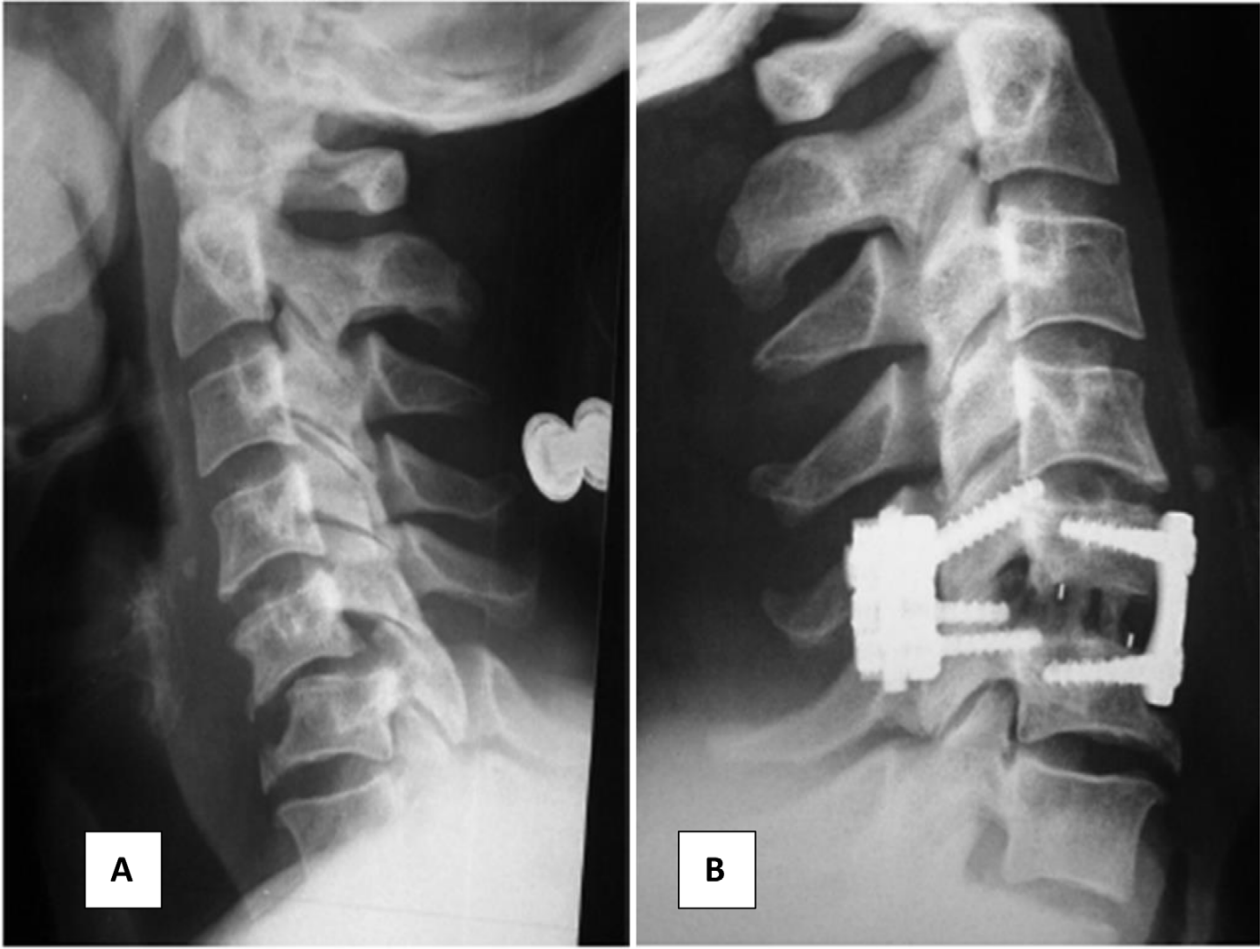
Irreducible neglected dislocation C6–C7 (slide A). Corpectomy C7 and fixation in situ using mesh cage and plate (slide B).

Conversely, corpectomy of the caudal vertebra and fusion in situ is an alternative technique for neglected lower cervical dislocations [16, 33]. It restored the alignment, and patients’ neurology improved significantly (Figure 6). Even after both posterior and anterior release, failure to achieve reduction may necessitate recourse to in situ fixation and fusion [30].

## Limitations

This review considered exclusively the English literature about neglected lower cervical facet dislocations that are mainly case reports and few retrospective studies. Being a narrative review, we cannot have a sound comparison of the techniques and approaches to these injuries. Most studies reported good outcomes and few postoperative complications. However, retrospective studies tend to underestimate spine surgery complications compared to prospective studies [45]. While most of the reviewed publications involved the Asian population, the occurrence of neglected lower cervical facet dislocations in other low- and middle-income countries (LMICs) is underreported, and we could not address them in this review.

## Conclusion

Delayed presentation of lower cervical facet dislocations remains a rare entity with notable controversies and variability around treatment techniques and approaches. Most patients are middle-aged adults, predominantly males. The most affected levels are C5–C6 and C6–C7. Delays are due to missed injuries, ineffective non-operative treatment, and negligence in seeking medical care. Patients present with variable degree of neurological deficits, persistent neck pain, and neck stiffness. Radiological assessment is important to delineate the injury morphology and changes related to the delay. Reduction of these injuries is challenging, and closed reduction is likely to fail. They required a variable combination of anterior and posterior surgical approaches for reduction and fixation. Reported approaches and techniques generally yielded improvement in pain, alignment, and neurology. The posterior approach aimed at surgical release and reduction while the anterior approach aimed at decompressing neural structures. Fusion in situ is an alternative to consider for failed reduction while conservative treatment may work for auto-fused cases. Given existing controversies, the need for quality prospective studies to determine the best treatment approach for lower cervical facet dislocations presenting with delay is evident.

## Conflict of interests

The authors declare that they have no relevant financial or non-financial interests to report.

## Funding

This research did not receive any specific funding.

## Ethical approval

The ethical approval was not required.

## Author contributions

L. Nkurikiyumukiza: Conceptualization, methodology, writing the original draft. A.M. Buteera: Supervision, reviewing, and editing. M.M. El-Sharkawi: Conceptualization, supervision, reviewing, and editing.
